# Changes in Composition and Function of Human Intestinal Microbiota Exposed to Chlorpyrifos in Oil as Assessed by the SHIME^®^ Model

**DOI:** 10.3390/ijerph13111088

**Published:** 2016-11-04

**Authors:** Julie Reygner, Claire Joly Condette, Aurélia Bruneau, Stéphane Delanaud, Larbi Rhazi, Flore Depeint, Latifa Abdennebi-Najar, Veronique Bach, Camille Mayeur, Hafida Khorsi-Cauet

**Affiliations:** 1 Laboratoire Périnatalité et Risques Toxiques (PERITOX), UMR-I01 INERIS, UPJV-CURS CHU Sud, Avenue Laennec, F-80054 Amiens CEDEX 1, France; julie.reygner@u-picardie.fr (J.R.); claire.joly@u-picardie.fr (C.J.C.); stephane.delanaud@u-picardie.fr (S.D.); veronique.bach@u-picardie.fr (V.B.); 2 UP-EGEAL 2012.10.101, Institut Polytechnique LaSalle Beauvais, 19 Rue Pierre Waguet, F-60026 Beauvais CEDEX, France; larbi.rhazi@lasalle-beauvais.fr (L.R.); flore.depeint@lasalle-beauvais.fr (F.D.); latifa.najar@lasalle-beauvais.fr (L.A.-N.); 3 Laboratoire Lymphocyte Normal, Pathologique et Cancer (LNPC), EA4666, UPJV-CURS CHU Sud, Avenue Laennec, F-80054 Amiens CEDEX 1, France; 4 INRA, UMR1319 MICALIS, AMIPEM, F-78350 Jouy-en-Josas, France; aurelia.bruneau@inra.fr; 5 AgroParisTech, UMR1319 MICALIS, F-78350 Jouy-en-Josas, France; camille.mayeur@inra.fr; 6 Commensal and Probiotics-Host Interactions Laboratory, INRA, UMR1319 MICALIS, F-78350 Jouy-en-Josas, France

**Keywords:** pesticide, microbiota, human fecal ecosystem, diversity, functional activity, SHIME^®^ model

## Abstract

The presence of pesticide residues in food is a public health problem. Exposure to these substances in daily life could have serious effects on the intestine—the first organ to come into contact with food contaminants. The present study investigated the impact of a low dose (1 mg/day in oil) of the pesticide chlorpyrifos (CPF) on the community structure, diversity and metabolic response of the human gut microbiota using the SHIME^®^ model (six reactors, representing the different parts of the gastrointestinal tract). The last three reactors (representing the colon) were inoculated with a mixture of feces from human adults. Three time points were studied: immediately before the first dose of CPF, and then after 15 and 30 days of CPF-oil administration. By using conventional bacterial culture and molecular biology methods, we showed that CPF in oil can affect the gut microbiota. It had the greatest effects on counts of culturable bacteria (with an increase in Enterobacteria, *Bacteroides* spp. and clostridia counts, and a decrease in bifidobacterial counts) and fermentative activity, which were colon-segment-dependent. Our results suggest that: (i) CPF in oil treatment affects the gut microbiota (although there was some discordance between the culture-dependent and culture-independent analyses); (ii) the changes are “SHIME^®^-compartment” specific; and (iii) the changes are associated with minor alterations in the production of short-chain fatty acids and lactate.

## 1. Introduction

In recent years, the intensive use of pesticides has become a worldwide public health concern. One of the most commonly used pesticides in agricultural and residential applications is chlorpyrifos (CPF), an inexpensive organophosphate compound that kills a broad spectrum of insects. Despite its now limited use in the USA and Europe, CPF is still widely used in other parts of the world, including Asia [1]. The widespread use of CPF leads to exposure through dermal, respiratory and oral routes. Residual CPF in food (especially in fruits and vegetables) at a level below that required for acute toxicity constitutes the main pathway for exposure [2,3]. CPF is a powerful, acutely neurotoxic compound that acts (through a CPF-oxon metabolite) by inhibiting acetylcholinesterase (AChE) in the nerve synapse. This results in functional changes in the organism’s tissues [4]. The clinical signs of acute pesticide poisoning in humans include tremors, eye tearing, headaches, nausea, behavioral disorders, stomach cramps, diarrhea and potentially life-threatening respiratory failure [5]. Nonetheless, several animal studies have shown that CPF exposure at levels below the threshold for systemic toxicity (inhibition of brain AChE) exerts disruptive effects on biological processes such as the cell cycle, apoptosis and DNA synthesis through non-cholinergic mechanisms [6,7]. Surprisingly, there are few animal studies of CPF’s effects on the gastrointestinal tract—the first organ to come into contact with food contaminants. In 2003, Cook et al. used a rat perfusion model to show that 99% of orally administered CPF is absorbed by the small intestine [8]. In 2012, another study indicated that CPF is biotransformed by cytochrome P450 in the upper part of the small intestine [9]. Lastly, Joly Condette et al.’s rat study showed for the first time that exposure to a low dose of pesticide causes morphological changes in the intestinal epithelium, increases intestinal permeability and bacterial translocation, and is associated with an imbalance in the intestinal microbiota [10,11].

The human gut is colonized by a broad variety of microorganisms, and disruption of the gut’s bacterial profile has often been linked to pathologies such as inflammatory bowel disease [12], obesity and diabetes [13,14]. The gut microbiota is a complex, dynamic ecosystem. It is composed mainly of bacteria but also comprises Archaea and yeasts. The bacterial count rises from the proximal colon to the distal colon, and reaches values of 10^11^ to 10^12^/g in feces [15]. The great majority of these bacteria are anaerobes from three main phyla: Gram-negative *Bacteroidetes*, Gram-positive *Firmicutes*, and Actinobacteria [16]. Among the *Firmicutes*, the butyrate-producing bacteria found in human feces belong to *Clostridium* clusters IV (*C. leptum*) and XIVa (*C. coccoides*) [17,18]. Gram-positive bifidobacteria and lactic acid bacteria (LAB, such as *Lactobacillus* spp.) are minor bacterial groups in humans but have a major role in human health [19]. The most distinctive benefits of bifidobacteria are modulation of host defense responses and protection against infectious diseases [20,21]. Fukuda et al. have shown that production of acetate by *Bifidobacteria* protects against *E. coli* infection [22]. Indeed, the gut microbiota in general has an important role in an individual’s health and well-being; it is involved in the host’s physiology, the regulation of metabolism and the extraction of energy from ingested food [23]. Thus, bacteria possess an arsenal of genes (the microbiome) encoding enzymes involved in nutrient fermentation. Dietary fibers are fermented by intestinal bacteria into short-chain fatty acids (SCFAs, such as butyrate, acetate and propionate) that have local and/or systemic effects [18,24].

Given the gut microbiota’s key role in host homeostasis, factors such as antibiotics [25,26], diet [27] and toxics (including pesticides) [10,11,28,29] can influence the intestine microbiota composition. In rodent models, chronic exposure to pesticide-contaminated foods leads to gut microflora dysbiosis; more specifically, the abundance of *Lactobacillaceae* fell significantly during CPF exposure [11,29]. Only two in vitro studies of the effects of low-dose CPF (mimicking chronic exposure) on the intestine have been published. Firstly, Tirelli et al. showed that CPF increases membrane permeability in an enterocyte cell culture model [30]. Secondly, Joly et al. used the in vitro Simulator of the Human Intestinal Microbial Ecosystem (SHIME^®^) to show that CPF exposure was associated with an increase in the total cultured bacterial count (a pattern associated with dysbiosis) [28]. In fact, CPF exposure resulted in an increase in the cultured *Enterococcus* spp. and *Bacteroides* spp. counts and a decrease in the number of lactic acid bacteria (LAB, such as *Lactobacillus* spp. and the bifidobacteria). The SHIME^®^ model is known to be a useful tool for studying: (i) interactions between microbiota; and (ii) the effects of prebiotics and other compounds on the microbial community’s composition and metabolic activities [31,32]. The SHIME^®^ has already been used to study the effect of prebiotics (such as fructooligosaccharide and inulin) on the fermentation pattern of the colon microbiota [33,34,35]. Microbiota interactions in this in vitro model have also been monitored, in order to investigate the relationship between the structure of gut microbial communities fed with different diets and their functional stability when challenged with antibiotics [32].

We used the SHIME^®^ in vitro model and molecular biology techniques to study the direct effects of below-threshold doses of CPF (1 mg/day, dissolved in rapeseed oil) on the composition, diversity and metabolic functions of the human fecal microbiota. Our data show that chronic CPF exposure (1 mg/day in oil) caused slight, transient changes in bacterial counts and fermentative activity, and shifted the human gut microbiota’s diversity profile.

## 2. Materials and Methods

### 2.1. Chemicals

Chlorpyrifos (*O*,*O*-diethyl, *O*-(3,5,6-trichloro-2-pyridyl)phosphorothioate, purity 99.8% ± 0.1%; LGC Standards, Molsheim, France) was dissolved in rapeseed oil (MP Biomedicals, Illkirch, France) and administered daily to the stomach compartment of the in vitro culture system at a dose of 1 mg/10 mL oil in a total feed volume of 200 mL (corresponding to the stomach compartment’s volume).

### 2.2. Description of the SHIME^®^

The SHIME^®^ is a dynamic, in vitro model that represents the different parts of the adult human gastrointestinal tract [36]. It is formed by six double-jacketed reactors simulating the stomach, the duodenum/jejunum, the ileum/caecum and the three segments (ascending, transverse and descending) of the colon (Table 1). All reactors were continuously stirred, held at 37 °C and kept under anaerobic conditions by flushing them with nitrogen for 15 min once a day. The last three reactors were maintained at physiological pH. Three times a day, 200 mL of SHIME^®^ feed and 90 mL of pancreatic juice (as described by [36,37]) were added to the stomach and duodenum/jejunum reactors, respectively. The last three reactors were inoculated with a mixture of fecal microbiota from four healthy adult volunteers, none of whom had suffered from gastrointestinal diseases or had taken antibiotics in the previous six months. Details of the inoculum have been published elsewhere [28]. Here, we describe a single “before-after” study in a single SHIME^®^. During the first two weeks of the experiment, control nutritional medium was added to the reactors; this enabled the bacterial community to adapt to the in vitro conditions and stabilize [38]. After this period, CPF 1 mg/day in oil was administered for 4 weeks. Samples were collected and analyzed at three time points: day D0 (after two weeks of microbiota stabilization and immediately before the first dose of CPF treatment) and after 15 (D15) and 30 (D30) days of treatment with CPF 1 mg/day in oil.

### 2.3. Plate Counts

The bacterial groups in liquid samples from each colon reactor (R4–R6) were analyzed on D0, D15 and D30, using specific media (Table 2). Ten-fold serial dilutions were prepared in Ringer’s solution (Oxoid, Basingstoke, UK). To provide technical replicates, two plates per dilution were inoculated with 0.1 mL of the two most appropriate sample dilutions. One plate was incubated at 37 °C for 48 h (to determine the aerobe count) and the other was incubated at 37 °C for 4 days in an anaerobic chamber (Bactron Anaerobic, Sheldon Manufacturing, Cornelius, OR, USA) to determine the anaerobe count. The “total bacteria” count corresponds to the sum of the log(CFU/mL) of total aerobes and the log(CFU/mL) of total anaerobes.

### 2.4. SCFA Assays

SHIME^®^ liquid samples from each colon reactor were collected and then frozen at −20 °C. Acetate, propionate, butyrate, valerate, caproate and branched SCFA concentrations were analyzed using gas-liquid chromatography after the aqueous extraction of acidified samples (Nelson 1020, Perkin-Elmer, St Quentin en Yvelines, France), as described by [39]. Gas chromatography was performed using a polyethylene glycol Nucol column (Supelco, Saint-Quentin Fallavier, France) under isothermal conditions in an oven at 100 °C, with a hydrogen flow rate of 10 mL/min, an on-column injector operating at 200 °C and an flame ionization detector operating at 240 °C. SCFA concentrations were expressed in mmol/L.

### 2.5. l- and d-Lactate Assay

l- and d-lactate levels were measured as described previously [40]. Briefly, SHIME^®^ samples were diluted in 0.1 M triethanolamine buffer (pH 9.15) and then precipitated with 6 N trichloroacetic acid (10%). l- and d-lactate levels in the supernatant were measured using an enzyme assay kit, according to the manufacturer’s instructions (Biosentec, Toulouse, France). The results were expressed in mmol/L.

### 2.6. Extraction and Amplification of DNA from SHIME^®^ Samples

Samples (0.20–0.25 mL) were collected from the three colon reactors on D0, D15 and D30, and immediately stored at −80 °C. Each sample’s total DNA was extracted as described by Godon [41]. The DNA was eluted in a final volume of 50 µL and stored at −20 °C. The DNA’s concentration and intactness were determined visually after electrophoresis on a 1% agarose gel containing ethidium bromide. Total bacterial DNA and *Bifidobacterium*-specific DNA were amplified using HotStar Taq DNA polymerase (Qiagen, Courtaboeuf, France) and specific primer sets and temperature-time programs (Table 3 and Table S1).

### 2.7. Temporal Temperature Gradient Gel Electrophoresis (TTGE)

PCR products were submitted to TTGE for sequence-specific separation using the DCode Universal Mutation Detection System (Bio-Rad, Paris, France), a 1 mm-thick, 16 × 16 polyacrylamide gel (as described by [42]) and 7 L of 1.25× Tris-acetate-EDTA electrophoresis buffer. The electrophoresis was performed at a fixed voltage of 63 V for 15 h, with an initial temperature of 66 °C and a ramp rate of 0.3 °C/h. The gel was stained in the dark by immersion for 30 min in a solution of SYBR Gold^®^ Nucleic Acid Gel Stain (Invitrogen, Eugene, OR, USA) and was then read on a Storm device (Molecular Dynamics, Bondoufle, France). The TTGE profiles were analyzed with GelCompar software (version 2.0, Applied Maths, Kortrijk, Belgium) in order to determine (i) the number of bands in each lane; and (ii) each band’s position and intensity. A principal component analysis (PCA) was used to identify patterns in data and to highlight similarities and differences. The degree of similarity between patterns was measured by calculating Pearson’s coefficient.

### 2.8. Real-Time Quantitative PCR (RT-qPCR) Analyses of Bacterial 16S rDNA Genes

Using the DNA extracted from SHIME^®^ samples, qPCR assays for specific bacterial 16S rRNA genes (with the primer and probes described in Table S1) [40] were used to determine the composition of the microbiota in each reactor on D0, D15 and D30. PCR inhibition was tested with DNA dilutions and the TaqMan exogenous internal positive control (Applied Biosytems, Carlsbad, CA, USA). No inhibition was detected in 1000-fold dilutions of the DNA; consequently, this dilution was used for all PCR amplifications. The RT-qPCR mixtures were amplified in an ABI PRISM 7000 system (Applied Biosystems). Total bacteria and the main bacterial groups (*Clostridium coccoides*, *Clostridium leptum*, *Bacteroidetes/Prevotella*, and *Bifidobacterium*) were detected using the TaqMan universal PCR system, whereas the subdominant bacterial groups (*Escherichia coli* and *Lactobacillus/Leuconostoc/Pediococcus*) were detected using the SYBR Green PCR system (Life Technologies, Saint Aubin, France). Standard curves generated from each specific reference strain [40] were used for quantification by plotting the cycle threshold (Ct) versus the cell count in the RT-qPCR system. The results were expressed in copy number of 16S rRNA gene per mL of microbioal biomass.

### 2.9. Statistical Analysis

Statistical analyses were performed with StatView software (version 5.0, Abacus Concepts Inc., Berkeley, CA, USA). Data were analyzed using non-parametric Kruskal-Wallis and Mann-Whitney tests. In all analyses, the threshold for statistical significance was set to *p* < 0.05.

## 3. Results

### 3.1. Bacterial Composition

**Figure 1:**
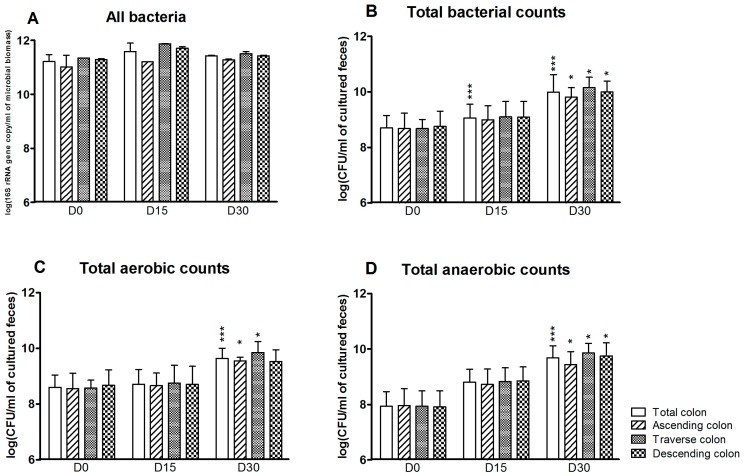
Total bacteria counts for the SHIME^®^’s colon as a whole and for each of the individual colon reactors at the end of the control period (D0) and after 15 and 30 days of exposure to pesticide chlorpyrifos (CPF) 1 mg/day in oil (D15 and D30). (**A**) Total bacterial counts determined by qPCR (Polymerase Chain Reaction) (log 16S rRNA gene copy/mL of microbioal biomass); (**B**) Total bacterial counts determined by plate culture (log CFU/mL of cultured feces); (**C**) Total cultured aerobe counts (log CFU/mL of cultured feces); (**D**) Total cultured anaerobe counts (log CFU/mL of cultured feces). Data are expressed as the mean ± Standard Deviation (SD) and were analyzed in a Kruskal Wallis test and then a Mann Whitney test. The level of statistical significance is indicated as follows: * *p* < 0.05; *** *p* < 0.001.

The effect of 1 mg/day CPF in rapeseed oil on the gut microbiota cultured in vitro was analyzed for the SHIME^®^’s colon as a whole and in each of the three individual reactors at D0 (before CPF-oil exposure), D15 and D30. Total bacterial counts and the specific bacterial profile were determined by plate counting and qPCR. According to the qPCR results, counts of “all bacteria” were high and stable in all three reactors and at all three time points. The mean counts ranged from 11.0 ± 0.4 to 11.9 ± 0.01 log(16S rRNA gene copy/mL of microbioal biomass). Treatment with CPF 1 mg/day in oil did not appear to have any effect on the total bacteria load (Figure 1A). In contrast, and although the counts were always lower than those determined by qPCR, the total bacteria load determined by plate counts increased during CPF 1 treatment (Figure 1B). Compared with D0, the total cultured bacterial count was significantly higher at D15 and D30 (*p* < 0.001) for the colon as a whole and at D30 for each of the colon compartments (*p* < 0.05) (Figure 1B). The mean cultured bacterial counts on D0 and D30 were 8.7 ± 0.13 and 9.9 ± 0.11 log(CFU/mL), respectively. Thus, after 30 days of CPF 1-oil mg/day administration, both cultured aerobe and anaerobe counts were above the D0 value in the simulated colon as a whole (*p* < 0.001) and in its individual segments (*p* < 0.05) (Figure 1C,D).

**Figure 2:**
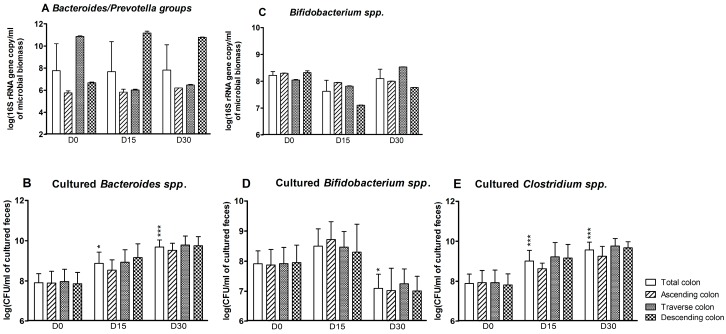
Anaerobe counts for the SHIME^®^’s colon as a whole and for each of the individual colon reactors at the end of the control period (D0) and after 15 and 30 days of exposure to CPF 1 mg/day in oil (D15 and D30). (**A**) *Bacteroides/Prevotella* groups determined by qPCR (log 16S rRNA gene copy/mL of microbioal biomass); (**B**) cultured *Bacteroides* spp. counts (log CFU/mL of cultured feces); (**C**) bifidobacterial counts determined by qPCR (log 16S rRNA gene copy/mL of microbioal biomass); (**D**) cultured bifidobacterial counts (log CFU/mL of cultured feces); and (**E**) cultured *Clostridium* spp. counts (log CFU/mL of cultured feces). Data are expressed as the mean ± SD and were analyzed in a Kruskal Wallis test and then a Mann Whitney test. The level of statistical significance is indicated as follows: * *p* < 0.05; *** *p* < 0.001.

According to the qPCR assays, the effect of CPF-oil treatment on *Bacteroides/Prevotella* groups at D15 and D30 varied from one colon reactor to another (Figure 2A). There was no change in the ascending colon reactor. However, CPF-oil exposure was associated with a reduction over time in the *Bacteroides/Prevotella* population in the transverse colon reactor (Figure 2A: D0: 10.9 ± 0.05; D15: 6.0 ± 0.1; D30: 6.5 ± 0.1 log(16S rRNA gene copy/mL of microbioal biomass)) and an increase in the descending reactor (D0: 6.7 ± 0.1; D15: 11.2 ± 0.2; D30: 10.8 ± 0.02 log(16S rRNA gene copy/mL of microbioal biomass)) (Figure 2A). Based on plate counts, the *Bacteroides* spp. population increased significantly in the colon as a whole at D15 (8.9 ± 0.2 (log CFU/mL of cultured feces; *p* < 0.05)) and D30 (9.7 ± 0.1 (log CFU/mL of cultured feces; *p* < 0.001)) (Figure 2B). In contrast, the molecular assay showed that exposure to CPF 1 mg/day in oil was associated with a light decrease in the bifidobacteria population in the colon as a whole at D15 only (D0: 8.2 ± 0.1; D15: 7.6 ± 0.4 log(16S rRNA gene copy/mL of microbioal biomass)). The impact was greater in the descending reactor at both D15 and D30 (Figure 2C). According to the culture method, a significant decrease in the bifidobacteria population was observed at D30 in the colon as a whole (D0: 7.9 ± 0.2; D30: 7.1 ± 0.2 (log CFU/mL of cultured feces; (*p* < 0.05))) (Figure 2D). In targeted molecular analyses, levels of the *Clostridium coccoides* and *Clostridium leptum* clusters were below the detection threshold—indicating that these extremely oxygen-sensitive bacteria were not alive (data not shown). In contrast, the *Clostridium* spp. count as determined by plate culture increased in the colon as a whole during CPF-oil exposure (D0: 7.9 ± 0.2; D15: 9.0 ± 0.2; D30: 9.6 ± 0.2 log CFU/mL of cultured feces; Figure 2E).

**Figure 3:**
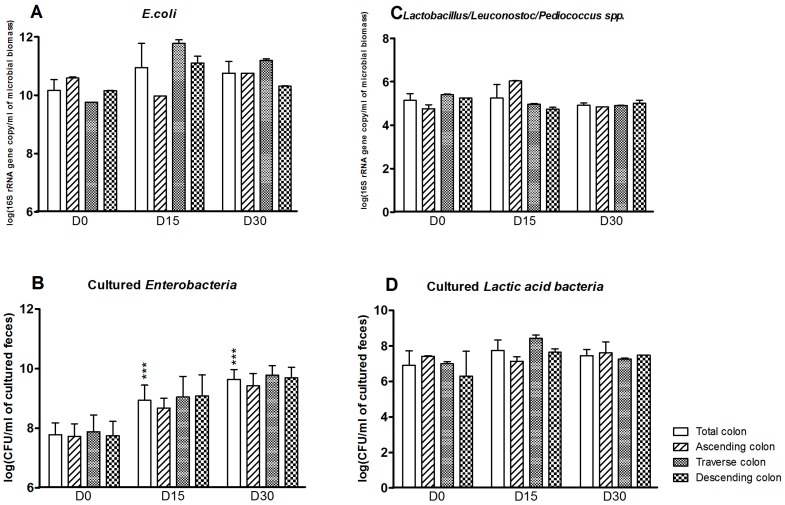
Aerobe counts for the SHIME^®^’s colon as a whole and for each of the individual colon reactors at the end of the control period (D0) and after 15 and 30 days of exposure to CPF 1 mg/day in oil (D15 and D30). (**A**) *E. coli* counts determined by qPCR (log 16S rRNA gene copy/mL of microbioal biomass); (**B**) Cultured enterobacterial counts (log CFU/mL of cultured feces); (**C**) *Lactobacillus/Leuconostoc/Pediococcus* counts determined by qPCR (log 16S rRNA gene copy/mL of microbioal biomass); (**D**) Cultured LAB counts (log CFU/mL of cultured feces). Data are expressed as the mean ± SD and were analyzed in a Kruskal Wallis test and then a Mann Whitney test. The level of statistical significance is indicated as follows: *** *p* < 0.001.

Some aerobic bacterial populations were also affected by exposure to CPF in rapeseed oil (Figure 3). The *E. coli* count was greater at D15 and D30 in the colon as a whole (D0: 10.2 ± 0.4; D15: 11.0 ± 0.8; D30: 10.8 ± 0.4 log(16S rRNA gene copy/mL of microbioal biomass)). The strongest effects were observed in the transverse and descending reactors (Figure 3A). The Enterobacteria plate counts (which target more genera—*E. coli*, *Klebsiella* spp., or *Citrobacter* spp., for example—than the molecular method) in the colon were significantly greater at D15 and D30 (*p* < 0.001) (Figure 3B). In contrast, exposure to CPF-oil treatment had no impact on the *Lactobacillus/Leuconostoc* counts as assessed by qPCR or plate counts (Figure 3C,D).

In summary, CPF-oil treatment affects the gut microbiota in vitro, although there is some discordance between the culture-dependent and culture-independent analyses. Exposure to 1 mg/day CPF dissolved in rapeseed oil was associated with a decrease in the colonic bifidobacterial population at D15 and an increase in the colonic *E. coli* count at D30 (according to qPCR data). Conventional plate culture techniques showed a rise in the total bacterial count, which reflected an increase in *Bacteroides* spp., *Clostridium* spp. and enterobacterial populations at D15 and D30 and a decrease in the bifidobacterial count at D30.

### 3.2. Bacterial Diversity

**Figure 4:**
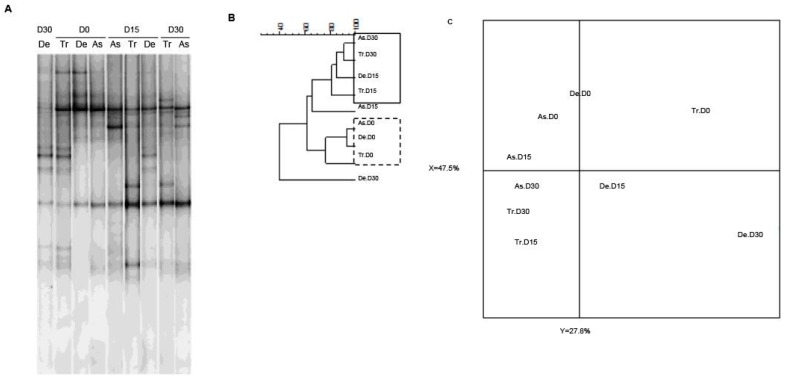
The Temporal Temperature Gradient Gel Electrophoresis (TTGE) fingerprint for total bacteria in the three colon reactors after 0, 15 and 30 days of exposure to CPF 1 mg/day in oil (D0, D15 and D30, respectively). (**A**) TTGE of all bacterial 16S rRNA gene amplicons in fecal samples collected from the colon vessels (As: ascending; Tr: transverse; De: descending); (**B**) The Pearson dendrogram for samples from each reactor at D0, D15 and D30; (**C**) The results of a PCA of samples from each reactor. The x and y values were 47.5% and 27.8%, respectively.

As slight quantitative differences in total bacteria and bifidobacterial counts were observed in the SHIME^®^’s compartments, we next sought to determine whether exposure to CPF 1 mg/day in oil was associated with changes in bacterial diversity. As previously demonstrated using two different techniques, the microbiota’s composition and diversity stabilize 2 weeks after starting up the SHIME^®^ [38]. Thus, we used PCR-TTGE to assess and compare the molecular compositions of microbial populations from the different colon compartments on D0, D15 and D30. Indeed, the fingerprint for total bacteria reflected an impact of CPF-oil exposure. On D0 (before CPF exposure), all three colon compartments had similar TTGE band profiles. The Pearson dendrogram and the PCA also showed that the bacterial compositions in the three colon compartments were very similar on D0 (Figure 4). In contrast, the TTGE profiles for D15 and D30 samples differed from those observed on D0 (Figure 4). The Pearson dendrogram and the PCA showed that samples at D15 and D30 were similar in molecular terms and therefore similar in terms of bacterial diversity. Indeed, the Pearson coefficients for the band patterns ranged from 74.8% to 93.4% on D0 and from 77.3% to 91.2% on D15 and D30 (Figure 4B). On D30, the descending colon’s diversity profile differed markedly from those in the two other reactors (Figure 4C).

**Figure 5:**
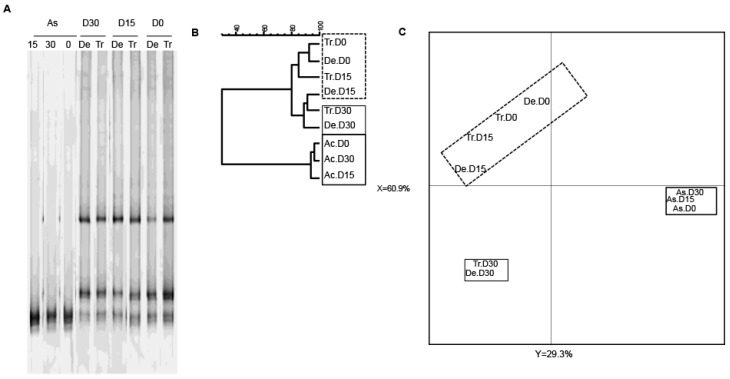
The TTGE fingerprint for bifidobacteria in the three colon reactors after 0, 15 and 30 days of exposure to CPF 1 mg/day in oil (D0, D15 and D30, respectively). (**A**) TTGE of bifidobacterial 16S rRNA gene amplicons of fecal samples taken from the colon vessels (As: ascending; Tr: transverse; De: descending); (**B**) The Pearson dendrogram for the reactor samples at D0, D15 and D30; (**C**) Results of a PCA of the samples from each reactor. The x and y values were 60.9% and 29.3%, respectively.

Given that bifidobacteria are an important bacterial group for human health, we next used a bifidobacterium-specific PCR assay to study the diversity of this group (Figure 5). Exposure to CPF 1 mg/day in oil was not associated with changes in the *Bifidobacterium* community (Figure 5A). Nonetheless, the TTGE data showed that the ascending colon reactor was less diverse than the transverse and descending colon reactors (Figure 5A). All the samples from the ascending colon clustered into one group, whereas the samples from the transverse and descending colon reactors clustered into another group (Figure 5B,C). The Pearson coefficients for the band patterns ranged from 93.1% to 96.4% for the ascending colon and from 70.7% and 92.6% for the other reactors. In a PCA, the gel patterns from the transverse and descending vessels differed from that observed in the ascending colon (at both D15 and D30), and thus reflected a slight effect of CPF-oil exposure on bifidobacterial diversity (Figure 5C).

In summary, exposure to CPF 1 mg/day in rapeseed oil altered the total bacteria diversity in the different vessels by D15, whereas the pesticide-oil’s effect on the bifidobacterial population in the transverse and descending colon reactors was only apparent on D30. These changes were relatively specific to the different colon compartments.

### 3.3. Levels of Bacterial Metabolites

**Figure 6:**
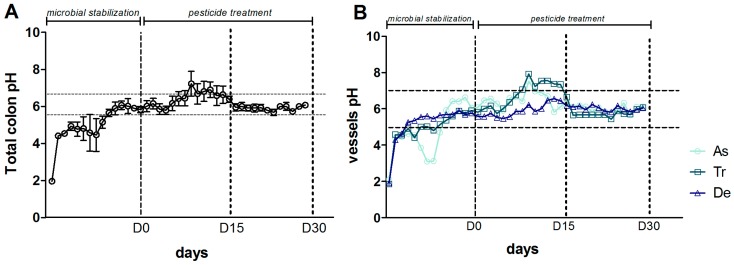
Change over time in the pH during the CPF1-oil treatment period (DO, D15 and D30) in (**A**) the colon as a whole (the mean value for the three colon reactors); and (**B**) each individual colon reactor.

An increase in pH during the control period (from D-14 to D0) and during the first 15 days of exposure to CPF-oil treatment (from D0 to D15) was observed in the SHIME^®^’s colon as a whole (Figure 6A) and in each of the three component reactors (Figure 6B). Between D15 and D30, all the pH values stabilized at around 6 (Figure 6A,B).

Exposure to CPF 1 mg/day in rapeseed oil did not have any significant effect on total SCFA production in any reactor or at any time point (Table 4). For the colon as a whole, exposure to CPF in oil was associated with a decrease in acetate and butyrate levels and an increase in propionate levels on D15; neither of these changes were statistically significant. However, the acetate and butyrate levels had essentially returned to their pre-exposure levels on D30. In the ascending colon, acetate production tended to increase gradually following CPF-oil exposure (D0: 14.2 ± 0.1; D15: 16.4 ± 1.1; D30: 18.2 ± 0.2 mmol/L). For the transverse and descending colon vessels, a large fall in acetate levels was seen at D15. However, this effect was only transient because the levels had fallen below the D0 value at D30 for the transverse colon (D0: 39.7 ± 0.02; D30: 35.1 ± 0.03 mmol/L) and had risen above the D0 value at D30 for the descending colon (D0: 27.0 ± 0.7; D30: 47.6 ± 0.02 mmol/L). Interestingly, CPF-oil treatment had the opposite effect on propionate production in the transverse and descending vessels. Indeed, it tended to decrease in the transverse colon and increase in the descending colon. The pesticide’s effect on butyrate production was similar in the transverse and descending colon reactors: a decrease in production at D15 and an increase at D30 were observed in both vessels.

Levels of branched SCFAs (protein metabolites) were also impacted by exposure to CPF 1 mg/day in oil. In the ascending and transverse reactors, the level had decreased by D15 but had returned to the D0 value by D30. In contrast, the level of branched SCFAs increased gradually in the descending colon reactor. The production of l-lactate decreased after 30 days of CPF-oil exposure (*p* < 0.01), whereas d-lactate levels increased significantly in the colon as a whole (*p* < 0.05) (Table 4). In the ascending colon, the l-lactate level increased on D15 (*p* < 0.05) and decreased at D30, while d-lactate level decreased until D30 (*p* < 0.05). In the transverse colon reactor, the l-lactate level decreased at D15 and increased at D30 (*p* = 0.06) while the d-lactate increased until D30 (*p* < 0.05). Lastly, in the descending colon reactor, levels of both l- and d-lactate falled at D15 and D30 (*p* < 0.05) (Table 4).

In summary, exposure to CPF 1 mg/day in oil slightly altered the fermentative activity of the human cultured microbiota. Firstly CPF 1 mg/day in oil modulated acetate, propionate and branched SCFA production in the transverse and descending colon vessels. Secondly, a low dose of CPF in oil mostly altered l- and d-lactate production in the ascending colon. l-lactate production peaked at D15 and fell by D30. In contrast, the d-lactate level increased with the CPF 1-oil treatment and peaked at D30 in the ascending colon vessel.

## 4. Discussion

Over the years, a range of in vitro models of the human intestinal tract have been developed [43,44,45]. In all these models, samples can be collected from each fermenter in order to investigate the exact location of metabolic transformations. However, these models only represent some of the specific physical aspects of the digestive tract and thus do not reflect the full range of physiological interactions between the microbiota and the host. Another limitation relates to the difficulty of obtaining sufficient replicates of a treatment under comparable conditions. Indeed, the best results are obtained if the same fecal slurry is used in all treatments. However, the burdensome nature of the equipment involved makes it difficult to run multiple systems in parallel with the same fresh fecal sample. One way of overcoming this problem (at least in part) is to sample multiple aliquots during each run. In contrast, major advantages of these in vitro systems include their similitude to the human in vivo context and the lack of a requirement for the ethical approval of experiments.

Our present results showed that 30 days of chronic exposure to a low dose of CPF (dissolved in oil) in an in vitro model of the human gut was associated with changes in the bacterial microbiota’s composition, diversity and fermentative activity. By applying conventional bacterial culture methods and molecular biology techniques, we found that the pesticide had a greater impact on viable/culturable bacteria than on total bacteria. The two methods gave different quantitative results. In general, conventional culture methods provide an estimate of the number of viable/culturable bacteria, which account for 20% to 40% of the intestinal microbiota [46]. Molecular techniques take account of the entire targeted bacterial population (which includes viable, non-culturable and inactive bacteria) and have a lower detection threshold than culture techniques. However, one inherent drawback of molecular methods (relative to culture techniques) relates to the use of specific primers and probes for a given bacterial group population. In contrast, molecular methods are able to detect unculturable or poorly culturable bacteria, such as the extremely oxygen-sensitive *Clostriduim leptum*. The concomitant use of these two approaches strengthened our data. For example, the MRS medium enabled the growth of LAB (such as streptococci and lactococci) as well as lactobacilli. This lack of selectivity might explain the two-log difference between the counts derived respectively by culture techniques and qPCR assays for this target population. Despite this difference, the results generated by the two techniques suggested that exposure to CPF-oil treatment did not have a significant impact on LAB in general and lactobacilli in particular. It has been reported that LAB isolated from the intestine [47] and from a Korean food preparation [48] were able to use CPF as a source of carbon and phosphate. Joly et al. (2013) reported a decrease in the number of lactobacilli in a similar in vitro model of the human gut [28]. However, the latter study only monitored lactobacilli, whereas the present study took account of all the LAB (given our use of MRS medium); this might explain the discrepancy between the two sets of results. Moreover, the studies showing that LAB use CPF as a carbon source were based on a simple model (isolated bacteria) or analyzed a food matrix; these are not dynamic, complex ecosystems like the one studied in the SHIME^®^. This aspect might explain the observed differences with regard to our results for the lactobacilli. In an earlier study, Joly et al., found that a decrease in the lactobacilli leads to a pH increase (a change from an acid pH to a basic pH) [28]. The dysbiosis favored other bacteria, such as *E. coli.* We nevertheless observed that the bifidobacterial count (as measured with conventional culture and a molecular method) fell after 30 days of CPF exposure, as previously reported by Joly et al. [28]. Since CPF is generally degraded by facultative aero-anaerobic bacteria [49,50], the increase in Enterobacteria counts (determined either by culture or a qPCR assay for *E. coli*) after 30 days of exposure might be a good marker of CPF metabolism. This increase might be stimulated by a CPF-induced rise in pH, since Enterobacteria are poorly tolerant of acidic conditions [51]. The 1-to-2 log difference in the counts determined with conventional vs. molecular techniques might be due to our choice of culture medium, which enabled the growth of all enteric bacteria. In contrast, our qPCR analysis of *C. coccoides* and *C. leptum* indicated that the numbers of these bacteria were below the detection limit (data not shown), whereas the conventional culture method revealed an increase in the population of clostridia after only 15 days of CPF exposure. The genus *Clostridium* includes a very large number of species, and the culture technique is less restrictive than the qPCR assay (which targets only two of the 19 *Clostridium* clusters) [52]. Regardless of these technical aspects, we found that chronic exposure to CPF-oil treatment induced a larger change in the population of viable/culturable bacteria. However, the changes in the bacterial community in the different parts of the in vitro intestinal model were also observed (albeit to a lesser extent) using molecular methods. As a consequence, we next determined whether CPF-oil treatment also impacted the diversity of the bacterial community. The TTGE molecular technique was used to monitor qualitative changes in bacterial composition in the different colon reactors and thus provided a dynamic overview of the diversity of the dominant bacterial species. A TTGE analysis of the total microbial population revealed an effect of CPF-oil exposure on total bacterial diversity, as indicated by the presence or absence of amplicons and differences in amplicon intensity in the different compartments of the simulated gut. On D0, the diversity was much the same in the three colon reactors, whereas the profiles of the ascending and transverse segments were relatively similar during the period of CPF-oil exposure. However, the diversity of the descending colon differed from that of the other two segments. Given that (i) quantitative analyses showed a decrease in the bifidobacterial count following CPF-oil exposure; and (ii) many bifidobacteria are classified as probiotics and have value in health outcomes, we also looked at the CPF treatment’s effect on *Bifidobacterium* diversity. The TTGE results depended on the compartment; there was less diversity in the ascending colon than in the transverse and descending segments. However, a PCA placed the transverse and descending segments in separate groups; 30 days of CPF-oil exposure appeared to have different effects on the ascending colon vs. the transverse and descending parts of the colon. The gastrointestinal tract’s microbial population has an important role in health because it modulates the colon’s digestive functions by producing SCFAs [53]. The measurement of metabolite levels provides important information on the microbiota’s functional status. An increase in pH was observed during the first 15 days of CPF-oil exposure. The subsequent slight fall in the pH might be due to the microflora’s metabolic adaptation to CPF exposure. The fermentation activity in the colon as a whole indicated that CPF-oil treatment had a transient inhibitory effect on acetate and butyrate at D15. Propionate and l-lactate levels increased at D15 and decreased at D30. l-lactate is mainly produced by *Bifidobaterium* and *Lactobacillus*, although the numbers of *bifidobacteria* (but not lactobacilli) fell during CPF-oil exposure. The gradual increase in the d-lactate level during CPF-oil exposure might be due to the increase in the *Bacteroides* count observed using culture methods. Indeed, *Bacteroides* spp. and lactobacilli are able to produce d-lactate [54]. In general, neither isomer is found in the feces of healthy individuals because both lactate isomers are either absorbed by colonocytes or metabolized into SCFAs by the gut bacteria [55,56]. The accumulation of d-lactate in feces reportedly reflects dysbiosis of the intestinal microbiota in short bowel syndrome [40]. The metabolic activity varied from one segment of the colon to another. For example, CPF-oil treatment has a very weak impact on bacterial fermentation in the ascending colon (relative to other colonic segments) which (due to the lower pH) is the main site of metabolic activity [51]. However, we observed a gradual increase in acetate and d-lactate levels and a decrease in the l-lactate level. The transverse colon had a very high pH over the first 15 days of CPF-oil exposure. This led to a transient decrease in fermentative activity and a drop in the release of l-lactate and the main SCFAs. However, the d-lactate level had increased in the transverse colon after 30 days of CPF-oil exposure. A high pH might explain the increase in *Bacteroides* spp. counts, as measured by plate culture [51]. Lastly, the descending colon seemed to be more metabolically active than the other segments, as reflected by an increase in acetate, propionate and branched chain fatty acids. However, the levels of l and d-lactate in this compartment fell—probably because lactates are intermediates in the production of SCFAs [40,57]. The concomitant increase in propionate levels and decrease in lactate levels might be due to the increase in numbers of lactate-consuming bacteria, such as *Eubacterium halli*, *Anaerostipes caccae*, *Coprococcus calus* and the clostridia (notably those from cluster IX, which are able to produce propionate from lactate [17,58]).

By using the SHIME^®^ model, we characterized the effect of chronic exposure to a low dose of CPF in oil on the human intestinal microbiota and on bacterial metabolism. Exposure to CPF in oil was associated with changes in several aspects of the intestinal microbiota (quantity, diversity and functionality). This model provides valuable information on intestinal microbial ecology, which is not addressed by other models (such as pure cultures) [59]. Given the current lack of in vitro models of bacterium-host cell interactions, it would be interesting to use other models to study the impact of pesticide exposure in a more integrated way [60]. Although the changes in our in vitro human model were not extremely pronounced, they do suggest that CPF has several effects on the human microbiota, and one can legitimately wonder whether these effects might be accentuated in immature organisms (such as newborns and infants). Indeed, Joly Condette et al.’s studies [10,11] showed that pesticide exposure had a greater effect (in terms of intestinal morphological changes, permeability and microbial imbalance) on rats during the weaning period than at adulthood. Hence, it would be very interesting to study the effects of pesticide exposure on a child’s intestinal microbiota by using a “Baby-SHIME^®^” model [43]. It now clear that in order to improve the assessment of toxic risks associated with environmental contaminants, we need to study the latter’s potentially harmful effects on the intestinal microbiota—a key “organ” for the individual’s health.

## 5. Conclusions

Chronic exposure to food contaminants (including pesticides) is now a major societal concern. Our results show for the first time that chronic exposure to CPF in oil can directly alter human microbiota in terms of quantity, diversity and metabolic activity. Our results may open up new perspectives for assessing the toxic effects of contaminants on the intestinal microbiota—a key “organ” in individual health. Studies of how pure cultures (a simplified system) might be affected by CPF could also provide mechanistic information on dysbiosis of the microbiota and possible impacts on human health.

## Figures and Tables

**Table 1:** Set-up of the SHIME^®^, with reactor volumes, residence times and pH values (based on [28]).

Reactor	Volume (mL)	Residence Time (h)	pH
R1: Stomach	200	3	2
R2: Duodenum/Jejunum	300	3	7
R3: Ileum/Caecum	300	4	7
R4: Ascending Colon	1000	20	5.5–6.0
R5: Transverse Colon	1600	32	6.0–6.4
R6: Descending Colon	1200	24	6.4–6.8

**Table 2:** Media and conditions used to determine counts of anaerobic and aerobic microbes.

Bacterial Group	Medium	Condition
**Total Aerobes**	Columbia Agar ^a^	Aerobic
**Total Anaerobes**	Blood Columbia Agar ^a^	Anaerobic
***Bacteroides*** **spp.**	Schaedler Agar ^a^	Anaerobic
***Clostridium*** **spp.**	Shahidi-Ferguson Perfringens ^a^	Anaerobic
**Enterobacteria**	Bromocresol Purple ^a^	Aerobic
**Lactic Acid Bacteria**	de Man Rogosa Sharpe ^b^	Aerobic
**Bifidobacteria**	Bereens	Anaerobic

^a^ Oxoid, Dardilly, France; ^b^ Sigma-Aldrich, Saint Quentin Fallavier, France.

**Table 3:** Primers and temperature-time programs used in the Polymerase Chain Reaction (PCR) amplification for Temporal Temperature Gradient Gel Electrophoresis (TTGE) diversity analysis.

Primer	Temperature-Time Program	Specificity
**Bact 968-GC-f** **Bact 1401-r**	(1) 15Ë′; 95 °C	Bacteria
	(2) 1′; 97 °C/1′; 58 °C/1′30; 72 °C (×30)	
	(3) 15′; 72 °C	
**Bif 164-f** **Bif 662-GC-r**	(1) 15′; 95 °C	*Bifidobacterium* spp.
	(2) 1′; 97 °C/1′; 58°C/1′30; 72 °C (×30)	
	(3) 15′; 72 °C	

**Table 4:** Concentrations of SCFA and l- and d-lactate in the colon as a whole and in each individual colon vessel for the control time point (D0) and during the treatment period (D15 and D30).

Vessel	Ascending			Transverse			Descending			Colon		
Time point	D0	D15	D30	D0	D15	D30	D0	D15	D30	D0	D15	D30
Total SCFA (mM)	15.3 ± 0.1	17.1 ± 0.3	19.3 ± 0.2	49.7 ± 0.1	6.6 ± 0.5	38.6 ± 0.1	29 ± 0.6	22.8 ± 0.003	58.7 ± 0.02	31.3 ± 12.3	15.7 ± 6.1	38.9 ± 8.9
Acetate	14.2 ± 0.1	16.4 ± 1.1	18.2 ± 0.2	39.7 ± 0.02	5.6 ± 0.5	35.1 ± 0.03	27 ± 0.7	14.7 ± 0.7	47.6 ± 0.02	27 ± 8.7	15.5 ± 4.4	33.6 ± 10.2
Propionate	0.8 ± 0.01	1.34 ± 0	0.9 ± 0.04	7.9 ± 0.01	0.9 ± 0.1	1.2 ± 0.001	0.8 ± 0.08	8 ± 0.07	9.2 ± 0.08	3.2 ± 3.1	4.7 ± 3.1	3.8 ± 3.6
Butyrate	0.1 ± 0.1	0.1 ± 0.0	0.1 ± 0.004	2 ± 0.03	0.1 ± 0.004	2.2 ± 0.03	1.2 ± 0.02	0.1 ± 0.01	1.8 ± 0.01	1.1 ± 07	0.1 ± 0.01	1.4 ± 0.8
Valerate	0.06 ± 0.01	0.0 ± 0.0	0.01 ± 0.0	0.1 ± 0.001	0.0 ± 0.0	0.0 ± 0.0	0.01 ± 0.0	0.0 ± 0.0	0.1 ± 0.02	0.05 ± 0.02	0.0 ± 0.0 *	0.1 ± 0.05
Caproate	0.08 ± 0.02	0.0 ± 0.0	0.02 ± 0.0	0.05 ± 0.01	0.0 ± 0.0	0.04 ± 0.003	0.04 ± 0.0	0.0 ± 0.0	0.04 ± 0.002	0.06 ± 0.01	0.0 ± 0.0 **	0.04 ± 0.001 **
Total Branched SCFA	0.1 ± 0.02	0.01 ± 0.01	0.07 ± 0.02	0.8 ± 0.01	0.0 ± 0.0	0.7 ± 0.01	0.1 ± 0.1	0.6 ± 0.001	0.7 ± 0.01	0.4 ± 0.3	0.3 ± 0.3	0.3 ± 0.004
l-lactate (mM)	1.4 ± 0.1	2 ± 0.1 *	0.3 ± 0.1 *	0.07 ± 0.0	0.0 ± 0.0	0.2 ± 0.03	0.5 ± 0.02	0.0 ± 0.0 *	0.0 ± 0.0 *	0.7 ± 0.5	2 ± 0.1	0.3 ± 0.1 **
d-lactate (mM)	1.7 ± 0.1	3.2 ± 0.1 *	7.3 ± 0.2 *	0.2 ± 0.1	0.2 ± 0.04	2.4 ± 0.1 *	0.6 ± 0.03	0.2 ± 0.1 *	0.5 ± 0.1 *	0.8 ± 0.6	1.2 ± 1.4	3.4 ± 2.6 *

Data are expressed as the mean ± Standard Deviation (SD). SCFA and lactate concentrations in the suspension (in mM). Values were compared in a Mann-Whitney test, SCFA = short-chain fatty acids. Signification * *p* < 0.05, ** *p* < 0.01.

## Supplementary Materials

The following are available online at www.mdpi.com/1660-4601/13/11/1088/s1. Table S1: Concentration of SCFA and l- and d- lactate in the colon as whole measured for the control time point (D0) and during the treatment period (D15, D30).
