# Supplementary material for: Changes in Composition and Function of Human Intestinal Microbiota Exposed to Chlorpyrifos in Oil as Assessed by the SHIME® Model
Source: Int J Environ Res Public Health. 2016 Nov 4;13(11):1088. doi: 10.3390/ijerph13111088 (PMC5129298; doi:10.3390/ijerph13111088)
Supplement: Supplementary file 1 [file ijerph-13-01088-s001.pdf]

Supplementary Materials: Changes in Composition and Function of Human Intestinal Microbiota Exposed to Chlorpyrifos in Oil as Assessed by the SHIME® Model

Julie Reygner, Claire Joly Condette, Aurélia Bruneau, Stéphane Delanaud, Larbi Rhazi, Flore Depeint, Latifa Abdennebi-Najar, Veronique Bach, Camille Mayeur and Hafida Khorsi-Cauet

Table S1. Concentration of SCFA and L- and D- lactate in the colon as whole measured for the control time point (D0) and during the treatment period (D15, D30).

System	**Target**	**Primers and Probe**	**Sequences (5′–3′)**
Taqman System	All bacteria	F-Bact 1369 R	CGGTGAATACGTTCCCGG
		Prok1492	TACGGCTACCTTGTTACGACTT
		P-TM1389F	6 FAM -CTTGTACACACCGCCCGTC
	C. leptum group	F-Clept09	CCTTCCGTGCCGSAGTTA
		R-Clept 08	GAATTAAACCACATACTCCACTGCTT
		P-Clep 01	6 FAM-CACAATAAGTAATCCACC
	C. coccoides group	F-Ccoc07	GACGCCGCGTGAAGGA
		R-Ccoc14	AGCCCCAGCCTTTCACATC
		P-Erec482	VIC- CGGTACCTGACTAAGAAG
	Bacteroides/Prevotella group	F-Bacter11	CCTWCGATGGATAGTGGTT
		R-Bacter 08	CACGCTACTTGGCTGGTTCAG
		P-Bac303	VIC-AAGGTCCCCCACATTG
	Bifidobacterium group	F-Bifid 09c	CGGGTGAGTAATGCGTGACC
		R-Bifid 06	TGATAGGACGCGACCCCA
		P-Bifid	6 FAM-CTCCTGGAAACGGGTG
Sybergreen System	*Lactobacillus/Leuconostoc/Pediococcus* group	F-lacto 05	AGCAGTAGGGAATCTTCCA
		R-Lacto 04	CGCCACTGGTGTTCYTCCATATA
	Escherichia coli species	F-Ecoli F	CATGCCGCGTGTATGAAGAA
		R-Ecoli R	CGGGTAACGTCAATGAGCAAA
	Target	Primers	Sequences (5′–3′)
TTGE PCR	*All bacteria*	Bact 968-GC-f	CGCCCGGGGCGCGCCCCGGGCGGGGCGGGGGCACGGGGG GAACGCGAAGAACCTTAC
		Bact 1401-r	GCGTGTGTACAAGACCC
	Bifidobacteria	Bif 164-f	GGGTGGTAATGCCGGATG
		Bif 662-GC-r	CGCCCGCCGCGCGCGGCGGGCGGGGCGGGGGCACGGGGG GCCACCGTTACACCGGGAA
